# Aims and Rationale of a National Registry Integrating Clinical, Echocardiographic, and Multi-Omics Profiling to Promote Precision Medicine in Peripartum Cardiomyopathy

**DOI:** 10.3390/biomedicines13082026

**Published:** 2025-08-20

**Authors:** Alessia Palmentieri, Ciro Battaglia, Dario D’Alconzo, Luigi Anastasia, Luca Bardi, Giuseppe Bifulco, Maria Calanducci, Martina Carotenuto, Paolo Ivo Cavoretto, Federica Carusone, Emilio Di Lorenzo, MariaFrancesca Di Santo, Attilio Di Spiezio Sardo, Federica Ilardi, Danila Ioele, Francesca Lanni, Marco Licciardi, Francesco Loffredo, Rachele Manzo, Daniele Masarone, Nicolò Montali, Roberta Paolillo, Vanessa Peano, Giovanni Peretto, Enrica Pezzullo, Pina Polese, Gabriele Saccone, Alaide Chieffo, Giovanni Esposito, Cinzia Perrino

**Affiliations:** 1 Department of Advanced Biomedical Sciences, School of Medicine, Federico II University, 80131 Naples, Italy; alessia.palmentierii@gmail.com (A.P.); cirobattaglia199@gmail.com (C.B.); dario.dalconzo@gmail.com (D.D.); marti.carotenuto@gmail.com (M.C.); federica.carusone@unina.it (F.C.); mariaf.disanto@gmail.com (M.D.S.); fedeilardi@gmail.com (F.I.); rachele.manzo@unina.it (R.M.); espogiov@unina.it (G.E.); 2 School of Medicine, Vita-Salute San Raffaele University, 20132 Milan, Italy; anastasia.luigi@hsr.it; 3 Institute for Molecular and Translational Cardiology (IMTC), IRCCS Policlinico San Donato, 20097 San Donato Milanese, Italy; 4 Department of Cardiovascular Sciences, Diagnostic Imaging and Time-Dependent Network of Cardiovascular Emergencies, Federico II University Hospital, 80131 Naples, Italy; luca.bardi@unina.it (L.B.); danilaioele11@gmail.com (D.I.); roberta.paolillo@unina.it (R.P.); pinapolese1@gmail.com (P.P.); 5 Department of Neuroscience, Reproductive Sciences and Dentistry, School of Medicine, Federico II University, 80131 Naples, Italy; giuseppe.bifulco@unina.it (G.B.); attiliodispiezio@libero.it (A.D.S.S.); gabriele.saccone.1990@gmail.com (G.S.); 6 Department of Gynecology and Obstetrics, School of Medicine, Vita-Salute San Raffaele University, 20132 Milan, Italy; calanducci.maria@hsr.it (M.C.); cavoretto.paolo@hsr.it (P.I.C.); 7 Department of Cardiology, AORN dei Colli-Monaldi Hospital, 80131 Naples, Italy; emidilorenzomd@gmail.com (E.D.L.); daniele.masarone@ospedaledeicolli.it (D.M.); 8 Department of Cardiology, AORN S. G. Moscati Hospital, 83100 Avellino, Italy; francesca.lanni@aornmoscati.it; 9 Interventional Cardiology Unit, IRCCS San Raffaele Hospital, 20132 Milan, Italy; licciardi.marco@hsr.it (M.L.); chieffo.alaide@hsr.it (A.C.); 10 Department of Cardiology, School of Medicine, University of Campania Luigi Vanvitelli, 81100 Caserta, Italy; francesco.loffredo@unicampania.it (F.L.); enrica_pezzullo@hotmail.com (E.P.); 11 Cardiology, Cardiovascular and Thoracic Department, Molinette Hospital, Città della Salute e della Scienza, 10126 Turin, Italy; nicolo.montali@gmail.com (N.M.); vanessapeano@gmail.com (V.P.); 12 Department of Cardiac Electrophysiology and Arrythmology, IRCCS San Raffaele Hospital, 20132 Milan, Italy; peretto.giovanni@hsr.it

**Keywords:** peripartum cardiomyopathy, metabolomics, transcriptomics, genomics, proteomics, precision medicine

## Abstract

**Background.** Peripartum cardiomyopathy (PPCM) is a rare but potentially life-threatening condition typically presenting as heart failure with reduced ejection fraction in the last month of pregnancy or in the first five months following delivery in women without other known causes of heart failure. PPCM incidence and prevalence are highly variable in different populations and geographical areas. The etiology of PPCM is likely multifactorial, with genetic predisposition, autoimmune conditions, nutritional deficiencies, hormonal and metabolic changes, myocardial inflammation, enhanced oxidative stress, vascular dysfunction, and angiogenic imbalance all listed as possible contributing factors. **Objectives.** The complexity and multifactorial nature of PPCM can be explored by large-scale “omics” investigations, and their integration has the potential to identify key drivers and pathways that have the largest contribution to the disease. The scarcity of relevant knowledge and experience with most rare diseases raises the unique need for cooperation and networking. **Methods and results.** In the context of PPCM, we hypothesize that the creation of prospective patient registries could represent an answer to this criticality. Therefore, we created a multicenter national registry of PPCM in different geographical areas in Italy. **Conclusions.** We expect that the integration of clinical, imaging and omics-based data might provide novel insights into PPCM pathophysiology and allow in the future early detection, risk assessment, and patient-specific therapeutic interventions, thereby offering new perspectives in precision medicine.

## 1. Introduction

**Figure 1:**
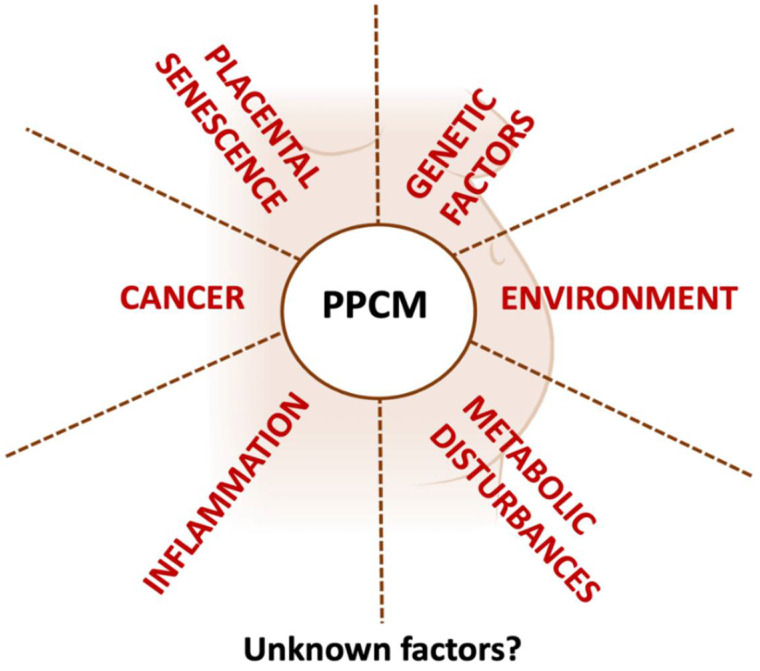
Schematic diagram representing known factors involved in PPCM pathophysiology.

According to the latest European Society of Cardiology (ESC) definition, peripartum cardiomyopathy (PPCM) is “a potentially life-threatening condition typically presenting as heart failure (HF) with reduced ejection fraction (HFrEF) in the last month of pregnancy or in the months following delivery in women without other known causes of HF” [1]. Despite this comprehensive definition, several clinical and scientific questions remain unanswered. First, while signs and symptoms of HF represent the major clinical findings necessary to suspect PPCM diagnosis, they need to be associated with a reduction in left ventricle ejection fraction (LVEF) with an arbitrary 45% cut-off value to support the diagnosis. Whether cases of HF with preserved ejection fraction (HFpEF) could be included is currently unknown. Secondly, although several clinical and preclinical studies have highlighted multiple possible pathophysiological determinants involved in PPCM (Figure 1), the current definition lacks any insights into the possible mechanisms responsible or involved in the development, progression, and prognosis of the disease. Etiology and mechanisms leading to the condition are crucial for a complete definition, hence the necessity to drive research in this direction.

Prospective registries represent an important step towards a deeper understanding of rare diseases such as PPCM. Thus, we created a national multicenter registry in Italy, providing clinical, echocardiographic and multi-omics profiling of enrolled PPCM patients. The primary objective of this experimental approach is to investigate the epidemiology, clinical, and imaging characteristics of these patients, and provide a multi-parametric phenotyping of PPCM patients, crucial to achieve precision medicine in this rare disease. Furthermore, the registry will document outcomes and monitor recurrence risk.

## 2. PPCM Clinical Characteristics

### 2.1. Epidemiology

PPCM is a rare disease whose incidence and prevalence are highly variable in different populations and geographical areas. The highest worldwide incidence of PPCM (1:102 deliveries) was found in Nigeria [2], while the lowest incidence (1:15,000 births) was recorded in Japan [3]. Incidence in remaining countries in Africa and Asia appears to be around 1:1000 births, while in the USA it ranges between 1:1000 and 1:4000 with some significant North/South differences [4,5]. The high incidence in Africa and Haiti confirms the higher risk in black women. However, it is unclear whether this high incidence is due to genetic background or to lifestyle, culture, socioeconomics, and/or accompanying pregnancy-related complications. In addition, PPCM in black women seems to have peculiar characteristics of presentation: a lower age of onset, with higher prevalence of hypertension and worse prognosis [6].

### 2.2. Risk Factors and Comorbidities

Risk factors for PPCM reported in different populations include age >30 years, black ethnicity, multiple gestation, obesity, positive family history of PPCM, and low socioeconomic status [7]. A strong correlation between gestational hypertension, preeclampsia, and PPCM has been reported [8], with twin pregnancies representing a significant risk factor for both PPCM and preeclampsia. Growing evidence suggests that PPCM and preeclampsia might share a similar pathogenesis, primarily driven by vascular abnormalities. The larger placental size in twin pregnancies might increase the secretion of antiangiogenic factors and inflammatory mediators, including interleukin-6 (IL-6), gamma interferon, and C-C Motif Chemokine Ligand 2/Monocyte Chemoattractant Protein-1 into the maternal circulation [9]. Soluble Fms-like tyrosine kinase-1 (sFlt-1), a molecule with antiangiogenic properties and potential cardiotoxic effects, that is increased in both conditions, may play a significant role [10].

### 2.3. Clinical Presentation

The possible clinical presentation of PPCM ranges from exertional signs and symptoms of HF to severe acute HF, pulmonary edema, cardiogenic shock, or even maternal sudden death [11]. Classic symptoms including dyspnea can easily mimic those described in physiological pregnancies. Other symptoms include cough, orthopnea, palpitations, chest and abdominal discomfort, swelling, paroxysmal nocturnal dyspnea, fatigue, malaise, dizziness, exercise intolerance, nocturia, and excessive weight gain. Key clinical signs include tachypnea, tachycardia, hypoxia, jugular vein distension, pulmonary rales or crackles, peripheral edema, displaced apical impulse, presence of an S3 heart sound, evidence of mitral or tricuspid regurgitation, hepatomegaly, and elevated jugular venous pressure.

Beyond the classic phenotype described above, PPCM can also present with arrhythmias or other complications related to reduced LVEF. Recorded arrhythmias cover the whole spectrum, with premature ventricular complexes as the most common manifestation, followed by ventricular fibrillation, non-sustained ventricular tachycardia, atrial fibrillation, and, more generally, conduction system disorders [12]. Physiological adaptations of pregnancy, such as the increase in blood volume and resting heart rate, might favor the development of rhythm disturbances. Alternatively, primary rhythm disturbances might be pre-existing and under-diagnosed. These conditions might lead to both benign arrhythmias and those potentially dangerous for pregnant women and the fetus [13]. Although benign arrhythmias such as sinus tachycardia, premature atrial contractions, and premature ventricular contractions are common during pregnancy, pathological atrial and ventricular rhythm disturbances are also on the rise, likely due to increasing maternal age and the growing burden of chronic diseases. Over the past ten years, the frequency of supraventricular tachycardia has remained stable, while the incidence of diagnoses of atrial fibrillation and ventricular tachycardia has increased, with atrial fibrillation now representing the most common clinically significant cardiac arrhythmia [14]. Cardiac arrhythmias are more common in patients with structural heart disease, both congenital and acquired, affecting between 5% and 9% of pregnancies in these cases [15]. Women with a previous history of cardiac arrhythmias have a recurrence risk of up to 52% during pregnancy and the postpartum period [16]. Embolic events are other forms of presentation, to which patients are particularly predisposed due to the hyper-coagulable state associated with pregnancy and the postpartum period, as well as the reduced ejection fraction [17].

### 2.4. Differential Diagnosis

PPCM is a diagnosis of exclusion. Therefore, differentiating it from other conditions that may present with similar signs and symptoms is of primary importance. These include primary dilated cardiomyopathy (DCM), pulmonary embolism, acute myocardial infarction, Tako-Tsubo syndrome, and pulmonary diseases. The diagnostic process relies on clinical evaluation, laboratory parameters, and, most often, electrocardiographic and echocardiographic assessment.

## 3. PPCM Etiology: An Ongoing Working Hypothesis

### 3.1. Cardiovascular Mechanisms Involved in PPCM

Pregnancy is characterized by remarkable hemodynamic changes, including the increase in blood volume, stroke volume, and reduction in peripheral vascular resistance leading to elevated end-diastolic volume and pressure, as well as left ventricular hypertrophy. These changes also affect heart rate and rhythm, increasing the risk of sinus tachycardia and arrhythmias. All these modifications usually occur in the first two trimesters, when patients with pre-existing heart disease tend to develop symptoms of HF [18]. Moreover, pregnancy is associated with a strong autonomic dysregulation with increased adrenergic tone, reducing coronary flow reserve, while increasing heart rate and cardiac workload. During pregnancy, a physiological increase in sympathetic tone at the expense of the parasympathetic tone is required to meet the increased hemodynamic and metabolic demands. However, when this process becomes dysfunctional, as in preeclampsia, the excessive rise in adrenergic tone leads to increased heart rate and, consequently, a higher risk of both minor and major arrhythmias. Hypertension develops, which in turn causes adverse remodeling of the left ventricle such as concentric cardiac hypertrophy [19].

Several factors have been involved in PPCM pathophysiology (Figure 1). A large amount of data suggests that placental senescence might contribute to PPCM as well as pre-eclampsia which, in turn, is promoted by a combination of placental and cardiovascular dysfunctions [20]. During gestation, the placenta produces and secretes various factors into the maternal circulation, like sFlt-1, a truncated, soluble form of Vascular Endothelial Growth Factor Receptor 1 (VEGFR-1) which serves as a circulating antiangiogenic protein secreted by the placenta especially in the last months of pregnancy, that binds and inhibits circulating Vascular Endothelial Growth Factor (VEGF) and Placental Growth Factor (PIGF) [21]. sFlt-1 is mainly responsible for hypertension and endothelial dysfunction in preeclampsia [22], and its levels are higher in women with PPCM [23]. While sFlt-1 decreases rapidly after delivery in healthy women, it remains high in women with PPCM [24].

Genetic factors are likely very important in PPCM development, affecting key structural and functional components of the cytoskeleton and ionic channels contributing to myocardial inflammation, HF, and arrhythmias [25]. In patients with genetic mutations, pregnancy could be seen as a “second hit” triggering the presentation of PPCM [26]. Electrolyte imbalances, in particular hypokalemia, which is quite common during pregnancy, might also increase the risk of HF, while predisposing patients to arrhythmias [27,28].

The highest incidence of PPCM in specific geographical hotspots such as Nigeria and Haiti support the hypothesis that a specific genetic background may underlie PPCM, although environmental factors might also be crucially involved. The recently concluded Peripartum Cardiomyopathy in Nigeria (PEACE) registry in Nigeria was a national consecutive study showing a significant association between Selenium deficiency, malnutrition, and PPCM, and that Selenium supplementation could be beneficial in the treatment of PPCM [2].

Significant metabolic dysregulation has also been demonstrated in PPCM, with an increased monocyte-to-High-Density Lipoprotein (HDL) ratio, elevated Low-Density Lipoprotein levels, increased adipogenesis, and nutritional deficiencies reducing contractility and promoting adverse ventricular remodeling. Notably, the monocyte-to-HDL ratio has been proposed as a novel marker associated with inflammation and oxidative stress. An increased ratio has been linked to adverse pregnancy outcomes, and in particular to persistent left ventricular dysfunction in patients with PPCM [29,30]. Hyperglycemic spikes contribute to oxidative stress, increased inflammatory response, and endothelial dysfunction. A recent study highlighted the relationship between the Stress Hyperglycemia Ratio (SHR)—a key factor in distinguishing chronic hyperglycemia from stress-induced hyperglycemia—and poor clinical outcomes in PPCM due to persistent left ventricular (LV) dysfunction [31].

The role of inflammation has been also intensely studied in PPCM patients, since they often display increased levels of Tumor Necrosis Factor α (TNFα), IL-6, soluble Fas/Apoptosis antigen 1(sFas/Apo1), and C-Reactive Protein (CRP) [32]. It has been also suggested that fetal cells translocation into the maternal circulation, while not exerting detrimental effects during pregnancy because of physiological immunosuppression, might induce an autoimmune response in the immediate postpartum period, as soon as the immune system has re-adjusted [24,33,34].

A link between PPCM and cancer has been also proposed, with a higher risk of cancer before and after PPCM diagnosis [35]. The type of cancer, cancer therapy, and/or specific gene variants, particularly those involved in DNA damage and repair, may connect PPCM and cancer [36,37].

### 3.2. Preclinical Models of PPCM

**Figure 2:**
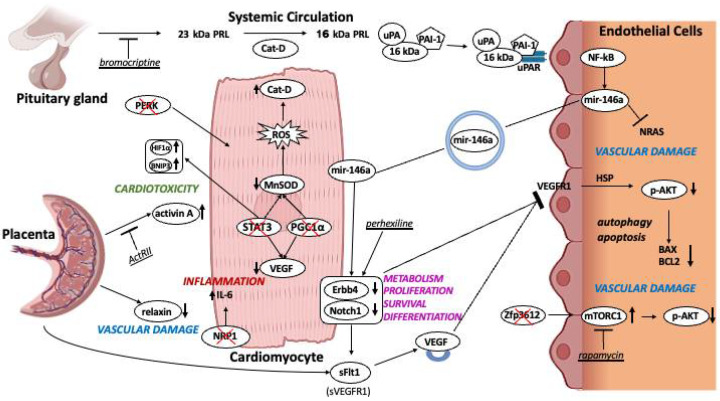
Schematic representation of major known signaling pathways involved in PPCM.

Several preclinical studies have investigated the possible molecular mechanisms underlying PPCM development and progression (Figure 2). Variations in serum levels of PIGF and sFlt-1 might promote endothelial dysfunction and imbalance angiogenesis, resulting in a higher risk of preeclampsia and end-diastolic pressure, impaired tissue repair, while coronary microvascular dysfunction contributes to ischemia and arrhythmias [23].

Reduced Signal Transducer and Activator of Transcription 3 (STAT3) and PPARγ-coactivator-1α (PGC1α) signaling induces a PPCM-like phenotype by mediating oxidative stress, angiogenic imbalance, and abnormal metabolic regulation (Figure 2). During pregnancy, STAT3 is normally phosphorylated in cardiomyocytes, and regulates the expression of antioxidant enzymes and proangiogenic factors. Murine models with cardiac-specific deletion of STAT3 (conditional knockout, cKO) develop cardiac hypertrophy and HF in the peripartum period. Furthermore, in cKO-PP females there is increased myocardial capillary density that is lost in the postpartum phase, indicating a key role of STAT3 in maintaining postpartum myocardial angiogenesis. Failure of this process leads to hypoxia as indicated by increased expression of the hypoxia marker genes Hypoxia-Inducible Factor 1α and Bcl-2 family protein BNIP3, and a decreased content of energy-rich phosphate, apoptosis, and, subsequently, HF [38]. Moreover, Stat3 deletion reduces the levels of the antioxidant manganese superoxide dismutase (MnSOD), causing an increase in reactive oxygen species, which induce the secretion of the peptidase cathepsin-D (Cat-D) by cardiomyocytes (Figure 2). Extracellular Cat-D can cleave the circulating nursing hormone prolactin (PRL) 23 kDa into a 16 kDa fragment inhibiting migration and cell cycle progression and promoting apoptosis in endothelial cells (ECs). Circulating 16 kDa-PRL can interact with plasminogen activator inhibitor-1 (PAI-1) and urokinase (uPA). The complex links uPA receptor (uPAR) on ECs’ surface, inducing Nuclear Factor kappa-light-chain-enhancer of activated B cells (NF-κB) activation and microRNA-146a (miR-146) expression with subsequent vascular damage [39]. MiR-146a can be secreted throughout exosomes into the circulation and absorbed by cardiomyocytes. STAT3 cKO mice with PPCM phenotype displayed increased cardiac miR-146a expression with coincident downregulation of metabolic activity and decreased expression of Erythroblastic leukemia viral oncogene homolog 4 (Erbb4), Neuroblastoma RAS viral oncogene homolog, and Neurogenic locus notch homolog protein 1 (Notch1) [40].

Cardiomyocyte-specific heterozygous deletion of Erbb4 induced PPCM associated with decreased levels of VEGF [41]. Meanwhile, pregnant wild-type mice treated with Notch1 inhibitor LY-411575 displayed postpartum ventricular dilatation, myocardial hypertrophy, and interstitial fibrosis and reduced myocardial angiogenesis; moreover, inhibition of Notch1 markedly increased Cat-D and sFlt-1, and reduced phosphorylated STAT3 and VEGF [42]. Blocking miR-146a with locked nucleic acids in mice improved cardiac function and capillary density, reducing fibrosis [40]. In these models, rescue treatment with bromocriptine, inhibitor of PRL secretion from the pituitary gland, preserved postpartum survival, cardiac function and angiogenesis, prevented cardiac fibrosis and apoptosis, inhibiting upregulation of miR-146a and capillary loss [38,40,43].

Relaxin-2 is a 6kDa peptide hormone structurally related to the insulin superfamily. Its serum levels rise in the first trimester of pregnancy and remain high until the end of pregnancy. Relaxin-2 acts as a potent vasodilator and promotes angiogenesis. Additionally, it helps to reduce oxidative stress, enhance myofilament activity in murine cardiac cells, and attenuate cardiac fibrosis. The STAT3 cKO model has also been used to study the role of the pregnancy hormone relaxin-2 on PPCM progression as Relaxin-2 serum levels tend to be lower in PPCM patients. High dose of recombinant relaxin-2 increased capillary density but did not improve cardiac function, fibrosis, or inflammation [44]. In the same animal model, β1-adrenergic receptor activation induced HF, attenuated by perhexiline through the up-regulation of the cardioprotective ErbB4 receptor and Glucose Transporter Type 4, preventing mitochondrial impairment and subsequent cardiomyocyte dysfunction and death [45,46].

Cardiomyocyte-specific deletion of PGC-1α in mice (PGC1α cKO) also induced PPCM [34,47]. PGC1α is a transcriptional regulator of oxidative metabolism and angiogenesis in many cell types, in part via expression and production of VEGF and upregulation of MnSOD [48]. PGC1α cKO mice also showed increased cardiac signaling of activin-A, another placental-derived hormone affecting cardiomyocyte function and contractility. Treatment with senolytic fisetin or a monoclonal antibody directed against the activin type II receptor similarly improved heart function, suggesting that PPCM could be ameliorated reducing pathological cellular senescence in the placenta [47].

Disruptions in protein folding and cell response to misfolding could also play a role in PPCM as shown by two recent preclinical studies [49,50]. Female transgenic mice with cardiac-specific expression of a mutant form of Heat Shock Protein 20 with transgenic S10F mutation showed extensive cardiomyocyte loss; depressed cardiac function after multiple pregnancies; increased apoptosis and autophagy by reduction of B-cell lymphoma 2/Bcl-2-associated X protein (Bcl-2/Bax) levels and Protein Kinase B (AKT) activity. This study tested the use of probenecid in PPCM, showing a decrease in mortality and hypertrophy; a reduction in apoptosis through higher Bcl-2/Bax levels; and increased levels of VEGF [50]. Reactive oxygen species (ROS) overproduction also causes the misfolding of proteins in the endoplasmic reticulum (ER); leading to ER stress [51]. ER sensor Protein Kinase RNA-like ER kinase (PERK) initiates the unfolded protein response. Mice models with cardiomyocytes specific deletion of PERK (PERK cKO) developed heart failure accompanied by a marked decrease in VEGF expression; upregulation of ROS levels; accumulation of unfolded proteins; and Stat3 and Erbb4 suppression.

Regression of pregnancy-induced cardiac hypertrophy has been linked to the signals mediated by mechanistic Target of Rapamycin Complex 1 (mTORc1) and AKT, with the uncontrolled growth of the heart halted by the regulation of mTORc1 activation by zinc finger protein 36-like 2 (ZFP36L2) [44,52]. Mice with cardiomyocyte-specific deletion of ZFP36L2 showed uncontrolled cardiac growth during pregnancy due to the upregulation of mTORc1 pathway by reducing AKT phosphorylation; its restoration with rapamycin, a mTORc1 inhibitor, improved cardiac function [52]. Another recent model of PPCM involved mice with total deletion of natriuretic peptide receptor 1, encoded by Npr1 gene. The model showed cardiac hypertrophy, fibrosis, and a high level of IL-6 [53]. Taken together, results from preclinical models suggest that PPCM is a syndrome with multiple causes, mainly related to vascular and hormonal abnormalities. Although Stat3 cKO and PGC1-α cKO models recapitulate PPCM phenotype, their limitation is the lack of cardiac function recovery after delivery. Improving the characterization of PPCM phenotypes and genotypes in humans, along with the development of suitable preclinical models, would greatly advance knowledge in this field.

## 4. Diagnostic Evaluation

### 4.1. Clinical Assessment and Electrocardiogram

Clinical assessment and electrocardiogram (ECG) are crucial to suspect PPCM diagnosis. While ECG usually reveals nonspecific changes, it is common to observe sinus tachycardia, supraventricular tachycardia (including atrial fibrillation and flutter), and abnormalities of the ST tract and the T wave, signs of left ventricular hypertrophy. Anteroseptal Q waves, branch blocks, QT elongation, QRS enlargement, and ventricular tachycardia have also been observed in the literature [27].

### 4.2. Laboratory Tests

Currently used laboratory tests cannot be conclusive for PPCM diagnosis. Unexplained increased levels of troponins and Brain Natriuretic Peptide/*N*-Terminal-proBNP (BNP/NT-proBNP), which do not change significantly in physiological pregnancies, can raise the suspicion of PPCM [54,55]. Effector of 16 kDa PRL, miR-146a, has been proposed as a promising potential diagnostic marker to distinguish between PPCM and other cardiomyopathies [40].

### 4.3. Cardiac Imaging

Current diagnosis of PPCM relies on clinical and imaging criteria, especially echocardiography and, only in selected cases, cardiac Magnetic Resonance Imaging (cMRI). Transthoracic echocardiography, by being easily accessible, is the reference method to support or exclude the diagnosis of PPCM, and many findings and parameters should be considered, including LVEF < 45%, diastolic dysfunction, right ventricular dilation, pulmonary hypertension, left atrial or bi-atrial dilatation, mitral or tricuspid insufficiency, or cardiac thrombi with or without left ventricular dilation.

Advanced methods such as cMRI can help in the diagnosis, especially in patients whose acoustic window is difficult. In these cases, cMRI can provide precise information on cardiac structure and function, adding a prognostic value in the assessment of PPCM [56]. Although cMRI should be safe in pregnancy, ESC clinical practice guidelines [1] discourage the use of gadolinium because the effects of its administration on the fetus are not well-established [57].

### 4.4. Large Scale Omics Fingerprinting and Biomarkers Discovery

Given the complexity and multifactorial nature PPCM, large-scale unbiased “omics” studies such as genomics, transcriptomics, proteomics, and metabolomics could play a key role in uncovering biological markers that improve diagnosis, prognosis, and treatment strategies. Although integrating multiple omics layers has the potential to reveal key molecular drivers and pathways contributing to the disease, only a limited number of studies have explored these approaches in relation to the PPCM phenotype so far.

Several genetic sequencing studies have focused on PPCM, often identifying overlaps with DCM. One study analyzing 67 gene regions in 469 patients found 70 rare truncating variants across 12 genes, including BCL2-associated athanogene 3 (BAG3), Desmin, Desmoplakin (DSP), Fukutin, Filamin C, Integrin-Linked Kinase, Titin (TTN), Myosin Heavy Chain 6 (MYH6), Myosin Heavy Chain 7 (MYH7), Plectin, Thymopoietin, and Vinculin (VCL), all of which are involved in cardiac contractility and cellular stress responses [58]. Similarly, in a cohort of 172 patients, sequencing of 43 gene regions revealed 26 rare truncating variants in 8 genes—TTN, DSP, VCL, MYH6, Tropomyosin 1, Synemin, Dystrophin, and Lysosomal Associated Membrane Protein 2—which are linked to sarcomere structure and cell–cell adhesion [59].

Smaller cohort studies (n = 19 and n = 41) have reported mutations in genes such as MYH7, Sodium Voltage-Gated Channel Alpha Subunit 5, Presenilin 2, MYH6, and Troponin T2, associated with cardiac electrical conduction and amyloid precursor protein processing [60,61]. A genome-wide association study also identified a single nucleotide polymorphism (rs258415) on chromosome 12 near the Parathyroid Hormone-Like Hormone gene, pointing to a potential role in regulating blood flow during pregnancy [61].

Family studies involving cases of both PPCM and DCM have shown a high frequency of TTN mutations, supporting the hypothesis of shared genetic pathways between the two conditions [62]. Key genes commonly involved also include BAG3, and Lamin A/C, reflecting a genetic architecture similar to other cardiomyopathies [63].

Gene expression profiling of myocardial tissue from 12 PPCM patients, compared with healthy controls and DCM patients, revealed over 1200 dysregulated genes (672 upregulated and 529 downregulated), primarily related to extracellular matrix organization, collagen biosynthesis, and antiviral immune response. Importantly, 209 genes were specifically dysregulated in PPCM compared to DCM, indicating a distinct transcriptional profile [64].

Proteomic studies have further highlighted molecular differences in PPCM. For example, Li et al. found significant changes in thousands of proteins in PPCM patients versus controls. Upregulated proteins included Myosin Heavy Chain 10, Collagen Type XVIII Alpha 1 Chain, Superoxide Dismutase 3, Hyaluronan-Binding Protein 2, Serpin Family A Member 4, Latent Transforming Growth Factor Beta Binding Protein 2, C2, Serpin Family C Member 1, Matrix Remodeling-Associated 7, Visinin-Like 1, and Phospholamban, while Tensin 3, MYH6, and ATPase Sarcoplasmic/Endoplasmic Reticulum Ca2+ Transporting 2 were among those downregulated. Enriched pathways included complement and coagulation cascades, actin cytoskeleton regulation, and protein processing in the ER, Mitogen-Activated Protein Kinase (MAPK), and Phosphoinositide 3-Kinase-Protein Kinase (PI3K)/AKT signaling. Downregulated pathways included those involved in cardiac muscle contraction, oxidative phosphorylation, calcium signaling, and thyroid hormone signaling [65].

In another proteomics study with 67 PPCM patients, 1959 proteins showed altered expression. Upregulated signaling pathways included Hippo, Transforming Growth Factor Beta, Janus Kinase-Signal Transducer, and Activator of Transcription, Hypoxia-Inducible Factor, Wingless-Related Integration Site, and PI3K-AKT, as well as immune-related processes like cytokine–cytokine receptor interaction and differentiation of helper T cells (Th) Th1/Th2/Th17. Comparison with non-peripartum cardiomyopathy revealed upregulation of Synaptogenesis, Soluble *N*-Ethylmaleimide-Sensitive Factor Attachment Protein Receptor, and Liver-X Receptor/Retinoid-X Receptor signaling, and downregulation of Rho GDP-Dissociation Inhibitor, BAG2, methionine degradation, and cytotoxic T-cell-mediated apoptosis [66]. Kodogo et al. also reported a set of differentially expressed proteins in PPCM, including upregulation of Adiponectin, Pregnancy-Specific Beta-1-Glycoprotein 1, Disintegrin and Metalloproteinase 12, Peptidylprolyl Isomerase A, Quiescin Sulfhydryl Oxidase 1, Fibronectin 1, Inter-α-Trypsin Inhibitor Heavy Chain, Fibulin 1, C7, C6, Serpin Family F Member 2, and downregulation of Ficolin-3, Pro-Platelet Basic Protein, Thrombospondin 1, Apolipoprotein A4, Apolipoprotein D, Haptoglobin, Selenoprotein P 1, Protein S1, Gelsolin, Antigen Presenting Cells System, Haptoglobin-Related Protein, Serpin Family G Member 1, Serpin Family D Member 1, and Hemopexin. The affected pathways included extracellular matrix remodeling, blood coagulation, immune responses, oxidative stress, and leukocyte migration [67].

Metabolomic analysis by Li et al. involving 7 PPCM patients found 29 significantly altered metabolites. Upregulated compounds were primarily nucleotide metabolism intermediates, while sugars and vitamin-related molecules were downregulated. Pathway analysis pointed to changes in arginine biosynthesis and mTOR signaling, while glucagon and Krebs cycle-related pathways were suppressed. Some of these findings overlap with those observed in DCM, suggesting shared mechanisms but also unique features in PPCM [65].

Finally, Fulghum et al. were among the first to apply a true multi-omics approach. In a mouse model of pregnancy-induced cardiac growth, they examined metabolic, transcriptional, and proteomic changes in the maternal heart. Their findings showed increased levels of metabolites like Pyruvate Dehydrogenase Kinase 4 and β-hydroxybutyrate Dehydrogenase 1 during late pregnancy, indicating a shift in cardiac metabolism away from glucose oxidation and toward alternative energy pathways [68].

## 5. Prognosis and Outcomes

The prognosis of PPCM is highly variable and depends on the severity of ventricular dysfunction at the time of diagnosis, response to therapy, and the presence of associated risk factors. The most significant prognostic indicator is LVEF as measured by transthoracic echocardiography. Patients with minimal reductions in ejection EF have a higher likelihood of complete recovery, whereas those with a reduction greater than 30% face a higher risk of adverse outcomes, such as thromboembolic complications, persistent HF, the need for heart transplantation, and mortality [69]. Persistent left ventricular dysfunction beyond six months is a crucial predictor of poor prognosis [70]. Patients with PPCM whose LVEF normalized have a better outcome; however, the residual cardiovascular risk remains higher than the general population. For this reason, both the ESC and the American Heart Association advise against subsequent pregnancies in women with PPCM who do not show EF improvement.

At the time of diagnosis, LVEF is the strongest predictor of adverse outcomes or long-term recovery [71]. Additional predictors of poorer outcomes include LV dilatation, thrombus, right ventricular systolic dysfunction, and obesity [72]. A strong correlation was observed between NT-proBNP levels over time and the New York Heart Association (NYHA) classification, as well as markers of inflammation and PRL levels. This suggests that NT-proBNP may be useful for monitoring ongoing inflammation or disease progression [73,74].

Prolonged QTc and sinus tachycardia at baseline ECG were independent predictors of poor outcome in PPCM after 6 months and 1 year, respectively [75]. A recent study highlighted the relationship between the SHR—a key factor in distinguishing chronic hyperglycemia from stress-induced hyperglycemia—and poor clinical outcomes. Furthermore, the study evaluated the predictive value of SHR for persistent LV dysfunction in patients with PPCM [31], suggesting that SHR could serve as a prognostic marker in this disease.

## 6. Rationale, Objectives, and Expected Outcome of a Multicenter National Registry Integrating Clinical, Imaging, and Multi-Omics Profiling of PPCM Patients

### 6.1. Rationale

Considering the young age of women affected by PPCM and the potential impact of the disease on their quality of life, morbidity, and mortality, additional research about prevalence, etiology, optimal therapy, long-term outcomes, and duration of treatment after recovery are of primary importance. For most rare diseases like PPCM, the scarcity of knowledge and experience from large scale clinical trials raises the unique need for cooperation and networking. In addition, clinicians with relevant expertise and adequate infrastructures to manage these patients are limited. Thus, the use of prospective patient registries is a critical first step in building a wide and comprehensive network for this heterogeneous disease, to better understand epidemiology, natural history, and molecular mechanisms and ultimately improve prognostic stratification and treatment. Importantly, because of knowledge gaps, the scope and objectives of rare disease registries are often broader than in a typical disease registry.

Between 2011 and 2018, the ESC EURObservational Research Programme PPCM (ESC EORP PPCM) registry enrolled more than 700 women with PPCM from approximately 50 countries [76]. Although the ESC EORP PPCM Registry primarily focused on clinical outcomes, serum from a subgroup of patients (n ≈ 84) identified altered expression of proteins related to autoimmune response and cardiac autoantibodies, supporting an immune-mediated component, coagulation pathways, with upregulation for example of fibrinogen, α_2_-antiplasmin, consistent with hypercoagulability in PPCM, and downregulation of mitochondrial and oxidative stress-related proteins (e.g., haptoglobin, hemopexin), highlighting impaired ROS scavenging and lipid metabolism [67]. A novel registry incorporating multi-omics profiling would represent a significant leap forward. By integrating genomics, transcriptomics, proteomics, and metabolomics with clinical phenotyping, such a registry could uncover the biological drivers of PPCM. This systems-level approach would help define molecular subtypes of PPCM, potentially distinguishing women with primarily genetic, inflammatory, or vascular forms of the disease.

In this context, we created a national multicenter registry in Italy under the supervision and coordination of Federico II University Hospital, Naples, Italy (NCT05878041) to address this criticality. Enrolled pregnant women with confirmed PPCM diagnosis undergo complete clinical, echocardiographic, and peripheral blood sampling to perform multi-omics profiling. Clinical management within centers of the network is coordinated by an interdisciplinary team of experts. By undertaking this approach, first we expect to significantly improve outcomes in patients with PPCM and, in the future and general perspective, of women with pregnancy- or sex-related cardiovascular disorders.

PPCM is a multifactorial disease with genetic, transcriptomic, proteomic, and metabolic alterations. Single-omics approaches might provide incomplete insight, often missing the dynamic interactions between molecular layers, while multi-omics integrating genomics (e.g., TTN and BAG3 mutations), transcriptomics (e.g., ECM and immune gene dysregulation), proteomics (e.g., altered coagulation and oxidative stress pathways), and metabolomics (e.g., disrupted energy metabolism) might enable the identification of disease-specific molecular signatures. This holistic approach might also distinguish PPCM from related cardiomyopathies like DCM, and highlight key regulatory pathways such as PI3K-AKT, MAPK, and mTOR. Multi-omics thus facilitates biomarker discovery and supports the development of personalized therapeutic strategies. By integrating clinical and multi-omics translational approach, we expect to provide a state-of-the-art study in-depth multiparametric phenotyping of patients with PPCM, providing novel important mechanistic information useful to achieve the unmet goal of personalized medicine in rare diseases.

### 6.2. Objectives

The major study objectives are the creation of a multicenter national registry of PPCM in different geographical areas in Italy, the collection of clinical, biochemical, and imaging information paired with biological samples, to assess incidence of this rare disease in different regions, associated comorbidities and risk factors, and gain novel in-depth information at multiple levels of biological and clinical testing.

After approval by the Institutional Ethics Committees, a pilot multicenter network with a hub/spoke structure with other hospitals in the city areas of the Units (Milan, Naples and Avellino) has been created.

After obtaining informed consent, patients with a suspected diagnosis of PPCM based on symptoms, risk factors, and comorbidities undergo clinical and echocardiographic screening. Once the PPCM diagnosis is confirmed according to the ESC guidelines: (i) development of the disease in the last month of pregnancy or within 5 months of delivery; (i) absence of an identifiable cause of HF; (iii) absence of recognizable heart disease before the last month of pregnancy; and (iv) LV systolic dysfunction demonstrated by classical echocardiographic criteria (LVEF < 45%), the patients are enrolled. Age-matched pregnant women at the same time of pregnancy, but without evidence of cardiovascular disease and HF, are enrolled as the control group. All enrolled patients undergo deep and multiparametric phenotyping through clinical, ECG, imaging, biochemical, and multi-omics (genomics, transcriptomics, metabolomics, and proteomics) assessments.

### 6.3. Study Procedures

**Figure 3:**
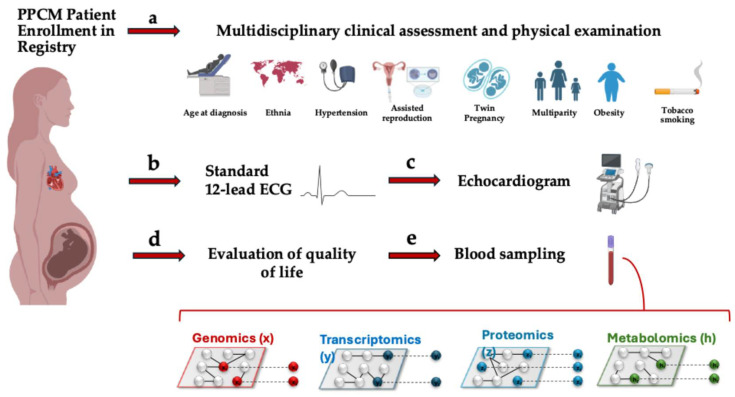
Schematic presentation of our systems biology approach to PPCM. After enrollment in the Registry, PPCM patients will undergo multidisciplinary clinical assessment, standard 12-lead ECG, echocardiography, evaluation of quality of life, and blood sampling to assess molecular signatures by multi-omics. Feature selection and predictive modelling will be used to investigate associations with disease severity, progression and recovery, as well as pathogenesis, biomarkers and drug discovery.

Enrolled patients with PPCM diagnosis will undergo clinical, biochemical, molecular, and imaging evaluations as depicted in Figure 3. At baseline we will perform the following:

(a) Multidisciplinary clinical assessment with extensive physical examination and patient interview, including medical and family history.
(b) Standard 12-lead ECG.
(c) Transthoracic echocardiography to primarily evaluate cardiac function. Whenever possible and if tolerated, cardiac magnetic resonance imaging will also be performed.
(d) Evaluation of quality of life by Kansas City Car diomyopathy Questionnaire and the EuroQoL (EQ-5D-5L) questionnaire.
(e) Blood sampling for standard laboratory tests, Peripheral Blood Mononuclear Cells (PBMC) isolation, and multi-omics profiling (whole exome sequencing; RNA sequencing; metabolomics; proteomics), as detailed in the next section.

The creation of an electronic case report form (eCRF) will ensure harmonization across centers by standardizing fields, formats, and validation rules. Centralized entry will minimize variability and enforce consistent terminology. Periodic audits will verify compliance, identify discrepancies early, and maintain high-quality, comparable data, strengthening the study’s future reliability and integrity.

### 6.4. Multi-Omics Strategy and Integration with Clinical Phenotyping

All biological samples will be collected in PPCM patients or in control subjects during pregnancy at an appropriate stage of gestation during enrollment or follow-up visits. For genomic analyses, whole exome sequencing will be performed on DNA extracted from whole blood collected in Ethylenediaminetetraacetic Acid (EDTA) tubes using the Illumina NextSeq 2000 platform (San Diego, CA 92122 USA). For transcriptomic profiling, total RNA will be extracted from blood collected in Tempus™ tubes (Thermo Scientific, Waltham, MA, USA) and processed using the Illumina Stranded Total RNA Prep with Ribo-Zero Plus Kit (Illumina, San Diego, CA, USA). Proteomic analyses will be performed on plasma extracted from EDTA blood using label-free quantitative mass spectrometry on a Thermo Fisher Orbitrap platform (Thermo Scientific, Waltham, MA, USA). Metabolomic profiling will be performed on plasma using untargeted liquid chromatography–mass spectrometry.

All pre-analytical procedures will be standardized throughout the network. Sample processing and storage will follow a common protocol under certified conditions to ensure sample traceability and integrity. Omics datasets will first be analyzed independently to identify rare variants, differentially expressed genes and proteins, and altered metabolic signatures associated with PPCM. The data will be then integrated using dimensionality reduction techniques (e.g., Principal Component Analysis), unsupervised clustering, and network-based algorithms, including multi-omics factor analysis and machine learning pipelines. All omics analyses will be performed within a core institutional infrastructure to ensure the highest level of data consistency and quality.

The integration of multi-omics and clinical data will be performed using machine learning algorithms to identify molecular patterns associated with disease severity and treatment response, enabling the development of personalized therapeutic strategies and risk prediction tools that improve clinical management and long-term outcomes. Validation will be performed at multiple levels: technical validation of key biomarkers (using quantitative Polymerase Chain Reaction and Enzyme-Linked Immunosorbent Assay), internal statistical validation (using cross-validation and training/test subsets), and biological validation (using concordance with existing PPCM and heart failure literature). This comprehensive approach aims to establish robust molecular signatures and identify potential biomarkers and therapeutic targets to improve clinical management and enable precision medicine in PPCM.

Validation of the multi-omics strategy will include replication in independent cohorts across multiple states in Europe through international registries, and through established biobanks with annotated clinical samples of patients with PPCM. Integrated analysis of genomics, transcriptomics, proteomics, and metabolomics with standardized phenotyping will be performed. Machine learning will ensure reproducibility, reliability, and predictive accuracy across diverse populations and datasets.

### 6.5. Follow-Up

All enrolled PPCM patients will be followed-up through a structured, multidisciplinary program involving cardiology, maternal-fetal medicine, and primary care. Early follow-up will include periodic echocardiograms, laboratory testing, and clinical evaluations at 3 and 6 months; then, patients will be followed-up annually at enrolling institutions. The centralized registry will document outcomes and monitor recurrence risk.

The registry’s long-term sustainability will be supported through an institutional backing. Ongoing data collection will be maintained via electronic health record integration, with built-in quality assurance protocols and routine audits to ensure data integrity. Dedicated personnel will oversee data governance and participant engagement. Future plans include integration with European and international networks to enable cross-border data sharing, harmonization of clinical and molecular data, and global collaboration on PPCM research.

### 6.6. Expected Outcomes, Scientific and Clinical Implications

PPCM remains a complex and multifactorial disease with unclear etiology and complex prognosis estimation, requiring advanced analytical approaches to guide early detection and precise risk estimation. Integration of clinical, imaging, and multi-omics-based data could allow the development of predictive models that go beyond traditional risk factors, offering more accurate tools to forecast recovery or recurrence. It might also create opportunities for identifying new therapeutic targets by revealing genetic mutations and dysregulated molecular pathways, paving the way for personalized treatment strategies. Coupling these molecular insights with a scalable registry infrastructure linked to biobanked samples and capable of harmonization with international datasets might position the registry not just as a tool for observation, but as a platform for discovery and innovation in PPCM research globally. An additional key long-term goal is to identify novel therapeutic targets or subsets of patients that might be more sensitive to already available therapies. For instance, bromocriptine may be more beneficial in patients with elevated PRL cleavage products or oxidative stress markers. Identifying such patient subgroups can allow tailored and precision therapeutic interventions.

Patients and clinical end-users will be actively engaged throughout the registry’s future implementations and dissemination phases. Clinicians will contribute to defining relevant clinical variables, workflows, and integration into practice. Regular stakeholder meetings and feedback loops will guide continuous improvement. Dissemination of results will include patient-friendly summaries, professional education materials, and policy briefs, ensuring the registry remains aligned with real-world needs and priorities.

## 7. Conclusions and Future Perspectives

PPCM remains a challenging and often devastating condition with variable epidemiology, largely unknown mechanisms, and unpredictable outcomes. The rarity of the condition, associated with limited mechanistic knowledge, forms the rationale for this study. The proposed national multicenter registry represents a transformative step toward understanding the complex interplay of clinical, imaging, and molecular factors in PPCM. By establishing an integrated infrastructure for data collection and analysis, the registry will facilitate breakthroughs in early diagnosis, risk stratification, personalized therapy, and long-term management. This initiative aims not only to advance the science of PPCM but also to provide tangible benefits to affected women and their families through improved care and outcomes. This registry will serve as a foundation for integrating clinical, imaging, and omics-based data. This could support earlier diagnosis and more effective therapeutic strategies, in line with the principles of precision medicine.

## Data Availability

The original contributions presented in this study are included in the article material. Further inquiries can be directed to the corresponding author.

## Abbreviations

The following abbreviations have been used in this manuscript:

PPCM	Peripartum Cardiomyopathy
ESC	European Society of Cardiology
HF	Heart Failure
HFrEF	Heart Failure with Reduced Ejection Fraction
LVEF	Left Ventricle Ejection Fraction
HFpEF	Heart failure with Preserved Ejection Fraction
IL-6	Interleukin-6
sFlt-1	Soluble Fms-like tyrosine kinase-1
DCM	Dilated cardiomyopathy
VEGFR-1	Vascular Endothelial Growth Factor Receptor 1
VEGF	Vascular Endothelial Growth Factor
PIGF	Placental Growth Factor
PEACE	Peripartum Cardiomyopathy in Nigeria
HDL	High-Density Lipoprotein
SHR	Stress Hyperglycemia Ratio
LV	Left Ventricle
TNFα	Tumor Necrosis Factor α
sFas/Apo1	Soluble Fas/Apoptosis Antigen 1
CRP	C-Reactive Protein
STAT3	Signal Transducer and Activator of Transcription 3
PGC1α	PPARγ-coactivator-1α
cKO	Conditional Knockout
MnSOD	Manganese Superoxide Dismutase
Cat-D	Peptidase Cathepsin-D
PRL	Prolactin
ECs	Endotelial Cells
PAI-1	Plasminogen Activator Inhibitor-1
uPA	Urokinase-Type Plasminogen Activator
uPAR	Urokinase-Type Plasminogen Activator Receptor
NF-κB	Nuclear Factor Kappa-Light-Chain-Enhancer of activated B cells
MiRNA-146a	MicroRNA-146a
Erbb4	Erythroblastic leukemia viral oncogene homolog 4
Notch1	Neurogenic locus notch homolog protein 1
Bcl-2/Bax	B-cell lymphoma 2/Bcl-2-associated X protein
AKT	Protein Kinase B
ROS	Reactive Oxygen Species
ER	Endoplasmic Reticulum
PERK	Protein Kinase RNA-like ER kinase
mTORc1	Mechanistic Target of Rapamycin Complex 1
ZFP36L2	Zinc Finger Protein 36-like 2
ECG	Electrocardiogram
BNP	Brain Natriuretic Peptide
NT-proBNP	N-Terminal-proBNP
cMRI	Cardiac Magnetic Resonance Imaging
BAG3	Bcl2-associated athanogene
DSP	Desmoplakin
TTN	Titin
MYH6	Myosin Heavy Chain 6
MYH7	Myosin Heavy Chain 7
VCL	Vinculin
MAPK	Mitogen-Activated Protein Kinase
PI3K	Phosphoinositide 3-Kinase-Protein Kinase
Th	Helper T cells
NYHA	New York Heart Association
ESC EORP PPCM	ESC EURObservational Research Programme PPCM
mTOR	Mammalian Target of Rapamycin
EDTA	Ethylenediaminetetraacetic Acid
